# Tackling malnutrition: a systematic review of 15-year research evidence from INDEPTH health and demographic surveillance systems

**DOI:** 10.3402/gha.v8.28298

**Published:** 2015-10-29

**Authors:** Samuelina S. Arthur, Bongiwe Nyide, Abdramane Bassiahi Soura, Kathleen Kahn, Mark Weston, Osman Sankoh

**Affiliations:** 1 INDEPTH Network, Accra, Ghana; 2 Department of Demography and Population Studies, University of the Witwatersrand, Johannesburg, South Africa; 3 Library Department, Systems and Technical Services, Mangosuthu University of Technology, Umlazi, Durban, South Africa; 4 Africa Centre for Health and Population Studies, University of KwaZulu-Natal, Durban, South Africa; 5 Ouagadougou HDSS, ISSP, University of Ouagadougou, Ouagadougou, Burkina Faso; 6 MRC Rural Public Health and Health Transitions Research Unit (Agincourt), School of Public Health, Faculty of Health Sciences, University of the Witwatersrand, Johannesburg, South Africa; 7 Umeå Centre for Global Health Research, Division of Epidemiology and Global Health, Department of Public Health and Clinical Medicine, Umeå University, Umeå, Sweden; 8 Independent Consultant, Mwanza, Tanzania; 9 School of Public Health, Faculty of Health Sciences, University of the Witwatersrand, Johannesburg, South Africa; 10 Faculty of Public Health, Hanoi Medical University, Hanoi, Vietnam

**Keywords:** nutrition, malnutrition, under and over nutrition, overweight, low- and middle-income countries, LMICs, health and demographic surveillance system

## Abstract

**Background:**

Nutrition is the intake of food in relation to the body's dietary needs. Malnutrition results from the intake of inadequate or excess food. This can lead to reduced immunity, increased susceptibility to disease, impaired physical and mental development, and reduced productivity.

**Objective:**

To perform a systematic review to assess research conducted by the International Network for the Demographic Evaluation of Populations and their Health (INDEPTH) of health and demographic surveillance systems (HDSSs) over a 15-year period on malnutrition, its determinants, the effects of under and over nutrition, and intervention research on malnutrition in low- and middle-income countries (LMICs).

**Methods:**

Relevant publication titles were uploaded onto the Zotero research tool from different databases (60% from PubMed). Using the keywords ‘nutrition’, ‘malnutrition’, ‘over and under nutrition’, we selected publications that were based only on data generated through the longitudinal HDSS platform. All titles and abstracts were screened to determine inclusion eligibility and full articles were independently assessed according to inclusion/exclusion criteria. For inclusion in this study, papers had to cover research on at least one of the following topics: the problem of malnutrition, its determinants, its effects, and intervention research on malnutrition. One hundred and forty eight papers were identified and reviewed, and 67 were selected for this study.

**Results:**

The INDEPTH research identified rising levels of overweight and obesity, sometimes in the same settings as under-nutrition. Urbanisation appears to be protective against under-nutrition, but it heightens the risk of obesity. Appropriately timed breastfeeding interventions were protective against malnutrition.

**Conclusions:**

Although INDEPTH has expanded the global knowledge base on nutrition, many questions remain unresolved. There is a need for more investment in nutrition research in LMICs in order to generate evidence to inform policies in these settings.

Nutrition has been defined as the ‘science of food, the nutrients and other substances therein, their action, interaction and balance in relation to health and disease, and the processes by which the organism ingests, absorbs, transports, utilises and excretes food substances’ (1).

In low- and middle-income countries (LMICs) studies of nutrition generally focus on malnutrition, defined by the World Health Organization (WHO) as ‘inadequate or excess intake of protein, energy and micronutrients such as vitamins, and the frequent infections and disorders that result’ (2). It is estimated that globally 2 billion people suffer from malnutrition (3), and this has been recognised as a leading cause of death, disability, and ill-health (4). Malnutrition is consequently the most important risk factor for the burden of disease in developing countries (5).

The long-term impact of malnutrition cannot be overemphasised (6–9). Such effects have severe consequences for individuals and families, dampening economic growth and poverty reduction. Currently, the commitment by developing countries and international bodies to address the problem of malnutrition – especially child under-nutrition – has never been higher (10, 11). Nutrition has consequently been elevated up the global development agenda, as the era of the post-Millennium Development Goals approaches. Synthesised research on key findings is needed to inform researchers and policy-makers of new evidence and knowledge as well as about neglected areas and gaps in nutrition research. This will help inform policy formulation aimed at addressing malnutrition. This paper highlights the contribution made by a longitudinal platform, the International Network for the Demographic Evaluation of Populations and their Health (INDEPTH), to nutrition research in LMICs.

The member centres of INDEPTH have played an important role in their efforts to measure the prevalence of nutritional disorders, understand their determinants and effects, and assess the effectiveness of interventions to tackle the problem (12). INDEPTH is a network of 49 health and demographic surveillance systems (HDSSs) based in Africa, Asia, and the Pacific region. Its member centres use longitudinal data, collected through regular visits to all households in a geographically defined area, to address the gaps in information on population health in LMICs. The HDSSs monitor new health threats; track population changes via fertility rates, death rates and migration; and measure the impact of policy interventions on communities. They aim to provide information that helps policy-makers to make informed decisions that adapt to changing conditions. While each centre contributes locally or nationally, as a network, INDEPTH has the potential to make global contributions.

In Africa and Asia, the HDSS centres have examined the problem of malnutrition using various methods, including cohort studies, nested surveys, case–control studies, qualitative focus group discussions, key informant interviews, literature reviews, clinical trials, and the testing of diagnostic tools. The centres have also developed and tested measurement and screening tools to facilitate a more accurate diagnosis of nutritional problems and to draw a more robust picture of food intake patterns in low- and middle-income settings.

## Methods

In this paper, we review studies of malnutrition conducted by HDSSs published in peer-reviewed English language journals, and we outline the key findings reported by INDEPTH member centres and discuss their implications for future nutrition policy. This study includes 67 published papers on malnutrition between 1998 and 2013, covering nine countries in Africa and four in Asia.

Standard systematic review methods by Higgins and Green (13) and the Centre for Reviews and Dissemination, York, United Kingdom, were used (14). Relevant publications titles (related to malnutrition studies) from the member research centres of INDEPTH were uploaded onto the Zotero research tool from different databases (60% from PubMed). Using the keywords ‘nutrition, malnutrition, and over and under nutrition’, publications were selected that were based only on data generated through research which used the longitudinal HDSS platform. All titles and abstracts were screened to determine inclusion eligibility and full articles were independently assessed according to inclusion/exclusion criteria. For inclusion, papers had to cover research on at least one of the following topics: the problem of malnutrition, its determinants, its effects, and intervention research on malnutrition. One hundred and forty eight papers were identified and independently reviewed by the authors and 67 were selected for this study.

**Fig. 1:**
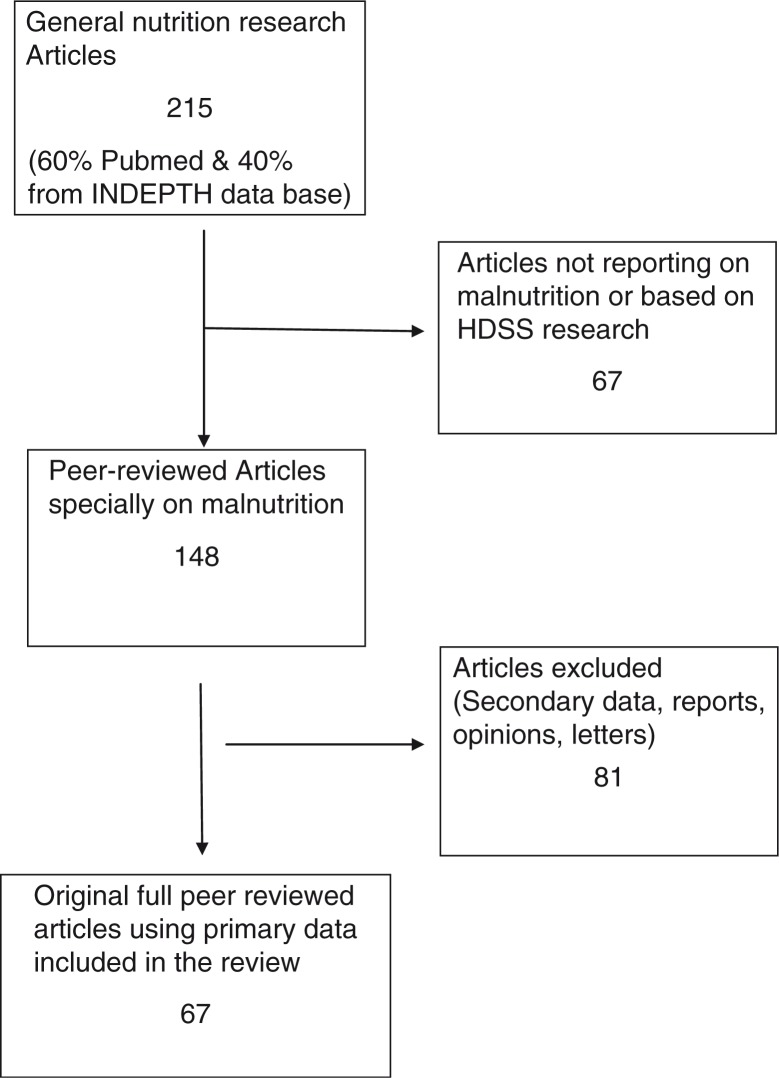
Diagram showing how the papers were selected for the study.

This paper deals with five tropical issues: The problem of malnutrition as manifested in member centres' demographic surveillance areas, including the prevalence of malnutrition and population groups most affected; the biological and social determinants of malnutrition; the effects of malnutrition; the interventions that have attempted to tackle malnutrition; and concludes with discussion and policy and research recommendations (Fig. 1).

## Results

### The problem of malnutrition

#### Under-nutrition

Insufficient or inadequate food intake – be it from lack of quantity or lack of essential nutrients including protein and specific micronutrients – remains a blight across the developing world. Under-nutrition can have effects throughout the life cycle. Physical stunting (defined by the WHO as low height-for-age) can affect cognitive development, increase susceptibility to infection, and impair school attainment and future productivity of survivors as well as of later generations. Deficiencies in iron, iodine, zinc, and vitamins can cause problems ranging from brain damage to blindness, anaemia, and stunted growth. Being underweight makes it harder to fight off infection and recover from illness, and harder to study and work.

Inadequate food intake in the early years of life is particularly damaging. In a cohort survey of children at Nouna HDSS in Burkina Faso, the authors found a reduction in underweight prevalence from 40% in June 1999 to 35% in June 2009 and from 43% in December 1999 to 34% in December 2009. The prevalence for wasting and severe wasting prevalence remained high in under-five children (15). In a 2010 study by Agincourt HDSS in rural South Africa, stunting was found in approximately 20% of children aged 1–4 years, and in almost one-third of those aged 1 year (16). In the same area in 2003/04, a case–control study, which aimed to determine risk factors for severe child malnutrition in Agincourt, found that 45% of controls under 5 years were stunted (17). Despite high levels of HIV in hospitalised cases, the persistence of more traditional risk factors indicated the need for interventions that concurrently target household socio-economic status, food security, education, feeding practices, and access to health and social services.

Being undernourished poses great risk for infants. A cohort study by researchers at the Nouna HDSS in Burkina Faso found that children aged between 6 and 30 months who were malnourished were more than twice as likely to die during the seven-month research period than other children (18).

Adult under-nutrition receives less attention than under-nutrition affecting children, but a population-based study at the Purworejo HDSS in Java, Indonesia, found that 15% of adult women were classified as suffering from chronic energy deficiency resulting from insufficient food intake (19). A later study by the same centre found that many women began pregnancy with suboptimal nutritional status and that most did not gain enough weight during pregnancy to avert the risk of adverse health outcomes (20). Malnutrition in pregnant women can pose problems not only for the women themselves but also for their newborns. A study in West Kiang HDSS, Gambia, showed that nutritional rickets, a disease caused largely by vitamin D deficiency, the effects of which can include cardiac failure and hypoglycaemic seizures, was more prevalent in infants whose mothers had poor vitamin D status during pregnancy (21).

A study of 457 people aged 60 years and above in rural Bangladesh found that 50% of older adults had chronic energy deficiency, and 62% were at risk of malnutrition (22). The authors argued that if global targets to reduce world hunger are to be achieved, ‘it is important to recognise that a substantial proportion of the elderly population, particularly in low-income countries, is undernourished’ (22).

#### Overweight and obesity

Under-nutrition is not the only form of malnutrition. Overweight and obesity, caused by an over-consumption of calories relative to energy expended, has long been considered a problem only in high-income countries, but in recent years it has begun to emerge as a threat in poorer countries.

As countries grow wealthier, the intake of refined and fatty foods and foods of animal origin increases. In 2002, the WHO ranked being overweight as the fifth most serious risk factor underlying the disease burden in low-mortality developing countries. INDEPTH member centres have begun to track the rise of obesity in developing countries over the past decade and a half. In the study of adult women by the Purworejo HDSS in Indonesia, researchers found an obesity rate in 1997 of 14% and showed that the rate had increased by almost three percentage points over the previous 12 months (19). Gendered patterns exist in rural South Africa. One study showed that females aged 50 years and older in Agincourt were significantly more obese than males, yet there was no difference in the prevalence of hypertension between men and women (23).

A 2005 study of 3-year-old children in the Limpopo province of South Africa found that 22% were overweight and 24% were obese. Nineteen percent of children were found to have both stunted growth and obesity (24). In Agincourt, rural South Africa, Kimani-Murage et al. (25) investigated adolescent weight status and central obesity (measured by abdominal circumference) and found much higher rates of combined overweight and obesity and of central obesity among girls (15% for each) than among boys (4% of whom had combined overweight and obesity, and 2% central obesity). In that study, risk factors for overweight and obesity included high socio-economic status and a household head who had completed secondary education. For central obesity, the risk factors included age, having a mother aged 50 years or above, and having high socio-economic status. A study at Kanchanaburi HDSS in Thailand found that urban residence, affluence, and possession of a television were associated with higher rates of obesity (26). Under-nutrition, on the other hand, was associated with poverty. The studies on over-nutrition point to the impact of modernisation in transforming lifestyles and suggest that as countries develop economically, the risk of obesity may increase.

Like under-nutrition, over-nutrition can also have harmful impacts on health. A cohort study of 1,888 rural women in Bangladesh found that those with the highest body mass index (BMI) had a higher mortality risk than those with intermediate BMI (27). Table 1 displays key findings on under-nutrition and obesity.

**Table 1:** Prevalence of under-nutrition and obesity: findings from INDEPTH studies

HDSS site	Year	Authors	Main findings
Nouna HDSS, Burkina Faso	2000	Beiersmann et al.	Slight reduction in the underweight prevalence from 40% in June 1999 to 35% in June 2009 and from 43% in December 1999 to 34% in December 2009.
Agincourt HDSS, South Africa	2010	Kimani-Murage et al.	20% of children aged 1–4 years had stunted growth.
Agincourt HDSS, South Africa	2007	Saloojee et al.	45% of children below the age of 5 had stunted growth.
Purworejo HDSS, Indonesia	2000	Winkvist et al.	15% of adult women had chronic energy deficiency from insufficient food intake; 14% were obese.
Matlab HDSS, Bangladesh	2006	Kabir et al.	50% of adults over 60 years of age had chronic energy deficiency; 62% at risk of malnutrition.
Agincourt HDSS, South Africa	2013	Gomez-Olive et al.	Women aged 50 years or above were significantly more obese than males.
Dikgale HDSS, South Africa	2005	Mamabolo et al.	22% of 3-year-old children were overweight or obese; 19% had both stunted growth and obesity.
Agincourt HDSS, South Africa	2011	Kimani-Murage et al.	15% of adolescent girls were overweight or obese, and 4% of adolescent boys.

### The determinants of malnutrition

#### Biological causes

Under-nutrition does not only come about because of lack of food. Exposure to disease can increase the risks posed to health by under-nutrition.

In a study at the Agincourt HDSS in South Africa, Kimani-Murage et al. (28) found that children infected with HIV had significantly worse nutritional outcomes than their uninfected peers. In Gambia, HIV-positive children had significantly worse scores in terms of weight-for-age, height-for-age, and BMI than HIV-negative children (29). In mothers, too, HIV poses problems. Research at the Africa Centre in Kwazulu-Natal found that breastfeeding mothers in South Africa who were HIV-positive had poorer protein and micronutrient status than HIV-negative women (30). Mean serum concentrations of albumin, pre-albumin, folate, retinol, and haemoglobin were all lower in the group of 92 HIV-positive breastfeeding women than in a control group of 52 HIV-negative women. Filteau et al. (31) found that providing pregnant women who are HIV-positive with vitamin A supplementation may improve the gut function of their HIV-infected infants. They also found that vitamin A supplementation for HIV-infected infants may decrease gastrointestinal morbidity.

Nyakeriga et al. (32) found a significant relationship between malaria and subsequent underweight and stunting in children below the age of 2 years at the Kilifi HDSS in Kenya. However, the authors found no association between malaria and subsequent protein-energy malnutrition. A Kisumu HDSS study in Kenya showed that infants who had had malaria were more likely to have stunting, anaemia, and iron deficiency (33). On the other hand, researchers at Farafenni HDSS in Gambia found no association between malaria and subsequent malnutrition in children below the age of 5 years during the malaria season (34). In Nouna, Burkina Faso, anaemia was significantly associated with malnutrition but not with the frequency of malaria episodes or its prevalence (35). A Kilifi HDSS study identified hospital-acquired bacteraemia (nosocomial bacteraemia) as a significant risk factor for severe malnutrition. Nosocomial infections, the study authors wrote, ‘are largely unrecognised or undocumented as a health risk in low-income countries, but they are likely to become public health priorities as awareness of their occurrence increases and as other prominent childhood diseases are progressively controlled’ (36). Table 2 displays key findings on biological causes of malnutrition.

**Table 2:** Biological causes of malnutrition: findings from INDEPTH studies

HDSS site	Year	Authors	Main findings
Agincourt HDSS, South Africa	2011	Kimani-Murage et al.	Children infected with HIV had significantly worse nutritional outcomes than their uninfected peers.
West Kiang HDSS, Gambia	2008	Nweneka	HIV-positive children had significantly worse scores in terms of weight-for-age, height-for-age, and BMI than HIV-negative children.
Africa Centre, South Africa	2007	Papathakis et al.	Breastfeeding mothers in South Africa who were HIV-positive had poorer protein and micronutrient status than HIV-negative women.
Africa Centre, South Africa	2001	Filteau et al.	Providing pregnant women who are HIV-positive with vitamin A supplementation may improve gut function of their HIV-infected infants. Vitamin A supplementation for HIV-infected infants may decrease gastrointestinal morbidity.
Kilifi HDSS, Kenya	2004	Nyakeriga et al.	Significant relationship between malaria and subsequent underweight and stunting in children below the age of 2 years.
Kisumu DSS, Kenya	2005	Mamiro et al.	Infants who had had malaria were more likely to have stunting, anaemia, and iron deficiency.
Farafenni HDSS, Gambia	2002	Deen et al.	No association between malaria and subsequent malnutrition in children below the age of 5 years during the malaria season.
Nouna HDSS, Burkina Faso	2003	Muller et al.	Anaemia was significantly associated with malnutrition.
Kilifi HDSS, Kenya	2011	Aiken et al.	Hospital-acquired bacteraemia (nosocomial bacteraemia) a significant risk factor for severe malnutrition.

#### Social causes

INDEPTH member centres have extensively investigated the social factors that increase the risk of malnutrition in LMICs. Three major contributors have been identified.

The first is wealth. Families themselves recognise this as important. Focus group discussions among mothers in Gambia revealed that poverty is one of the key constraints preventing mothers from putting into practise their knowledge of child health and nutrition (37). In similar discussions in Kenya, mothers reported that financial constraints were the main cause of severe malnutrition in children, with mothers’ heavy workload and inability to generate income were also reported as key factors. Witchcraft and the violation of sexual taboos were among the other reported causes (38). Household wealth and community socio-economic status were significantly associated with childhood stunting in Nairobi, Kenya (39). The authors of this study posited that the community effect occurs because of the availability of social services, physical environment, and the wealth of individuals. In an earlier paper, the same authors showed that neighbourhood characteristics exert an influence on malnutrition independently of household wealth – ‘when basic socio-economic and health services are lacking in the poorest communities’, they observed, ‘families therein can hardly take advantage of their increased means, ability and knowledge in caring for their children’ (40). In Agincourt, rural South Africa, cases with severe malnutrition were from households with fewer assets and lower incomes, who were less likely to receive a social support grant or to have anyone employed, compared with control groups in the village (17). In the same area, Kimani-Murage et al. (25) observed four-fold higher odds of overweight for adolescent girls and two-fold higher odds for participants from households with the highest socio-economic status. The causes of childhood malnutrition have been studied more extensively than those of adult malnutrition, but there is evidence that wealth continues to play a part in nutritional status later in life. As a 1998 study at Matlab HDSS in Bangladesh showed, women aged 35 years and older from wealthier households were less likely to have chronic energy deficiency than poorer women (41).

Education is a second key factor. Children born to Nairobi mothers who had been educated to primary level had a 94% lower chance of stunting than those born to mothers with no education (42). In one study in Vietnam, birth weight and growth were statistically significantly and positively associated with economic conditions and the education of the mother (43). In another study however, mothers’ education level was associated with their children's stunting in early childhood (44). As with wealth, education continues to have repercussions for nutritional status later in life – in the above-mentioned study in Bangladesh, women over the age of 35 were half as likely to suffer chronic energy deficiency if they had had one or more years of education, than if they had had no schooling (41).

Linked to both wealth and education is the associated process of urbanisation. Five of the nutrition studies carried out by INDEPTH member centres between 1998 and 2013 demonstrate a link between urban residence and reduced malnutrition. In a study in FilaBavi, Vietnam, Nguyen et al. (45) found an association between household socio-economic status, education of the mother and birth weight. The Nairobi study (39) discussed above confirmed this finding, with urban children significantly less likely to be malnourished than rural children, while two studies in Senegal showed that rural adolescent girls who migrated seasonally to urban areas for work had improved nutritional status, as reflected in BMI, compared with girls who remained in villages (46, 47). Although urbanisation appears to reduce the risk of under-nutrition, it may increase that of obesity. A study at Kanchanaburi HDSS in Thailand found that urban residence was associated with lower rates of under-nutrition in children, but also with higher rates of obesity (26). Household wealth was also associated with obesity.

One additional, yet less extensively studied social factor, which also appears to have an impact on nutrition, is paternal involvement in caring for children. Children in a study in South Africa whose fathers did not provide financial support were found to be at higher risk of malnutrition (48), while in Hanoi, Vietnam, children whose fathers took them to a medical facility to be immunised against childhood diseases were 1.7 times less likely to be underweight and stunted than other children (49). These points to the need for a multi-pronged response to the problem of malnutrition which has many causes (Table 3).

**Table 3:** Social causes of malnutrition: findings from INDEPTH studies

HDSS site	Year	Authors	Main findings
West Kiang HDSS, Gambia	2010	Mwangome et al.	Poverty a key constraint preventing mothers from putting into practise their knowledge of child health and nutrition.
Kilifi HDSS, Kenya	2011	Abubakar et al.	Mothers reported that ‘financial constraints’ were the main cause of severe malnutrition in children.
Nairobi HDSS, Kenya	2006	Fotso et al.	Household wealth and community socio-economic status significantly associated with childhood stunting.
Nairobi HDSS, Kenya	2005	Fotso et al.	Neighbourhood characteristics exert an influence on malnutrition independently of household wealth.
Agincourt HDSS, South Africa	2007	Saloojee et al.	Cases with severe malnutrition were from households with fewer assets and lower incomes that were less likely to receive a social support grant or to have anyone employed.
Agincourt HDSS, South Africa	2011	Kimani-Murage et al.	Four-fold higher odds of overweight for adolescent girls and two-fold higher odds for participants from households with the highest socio-economic status.
Matlab HDSS, Bangladesh	1998	Ahmed et al.	Women aged over 35 years from wealthier households were 0.77 times less likely to have chronic energy deficiency than poorer women.
Nairobi HDSS, Kenya	2011	Abuya et al.	Children born to mothers educated to primary level had a 94% lower chance of stunting than those born to mothers with no education.
Multiple sites in Ethiopia, India, Peru, Vietnam	2013	Barnett et al.	Mothers’ education level associated with stunting in early childhood of their children.
Matlab HDSS, Bangladesh	1998	Ahmed et al.	Women aged over 35 were half as likely to suffer chronic energy deficiency if they had had one or more years of education than if they had had no schooling.
Nairobi HDSS, Kenya	2006	Fotso et al.	Urban children significantly less likely to be malnourished than rural children.
Niakhar HDSS, Senegal	1999	Bénéfice et al.	Rural adolescent girls who migrated seasonally to urban areas for work had improved nutritional status compared with girls who remained in villages.
Niakhar HDSS, Senegal	2001	Garnier and Bénéfice	Rural adolescent girls who migrated seasonally to urban areas for work had improved nutritional status compared with girls who remained in villages.
Kanchanaburi HDSS, Thailand	2011	Firestone et al.	Urban residence associated with lower rates of under-nutrition in children, but also with higher rates of obesity.
Agincourt HDSS, South Africa	2007	Madhavan and Townsend	Children whose fathers did not provide financial support were at higher risk of malnutrition.
Chililab HDSS, Vietnam	2008	Tran et al.	Children whose fathers took them to a medical facility to be immunised against childhood diseases were 1.7 times less likely to be underweight and stunted than other children.

### The effects of malnutrition

Obesity is a relatively new threat in LMICs and is, as yet, not generally widespread in these countries. Therefore in researching the effects of malnutrition, HDSS centres have largely focused their attentions on the problem of under-nutrition.

A study by the Kilifi HDSS in Kenya found that among children admitted to a rural district hospital, under-nutrition was a factor in half the in-hospital deaths and half the morbidity from severe diseases such as malaria, gastroenteritis, lower respiratory tract infection, HIV, and invasive bacterial disease (50). At West Kiang in Gambia, young adults born in the ‘hungry season’ – that is, the non-harvest season between July and December when less food is available – had 10 times higher mortality than those born in the harvest season (51). Conversely, two studies at Institut de Recherche pour le Developpement IRD HDSS in Senegal found that although child mortality in the rural Casamance region declined sharply between 1969 and 1992, infants’ nutritional status did not improve over the period, suggesting other factors such as vaccination were more important (52, 53).

As well as increasing mortality, under-nutrition also impairs the development of those who survive it. In a study in Bangladesh, stunting in early childhood, which is in part the result of a combination of disease and under-nutrition, was found to be a significant predictor of stunting in adolescence (54). A four-country study covering Ethiopia, India, Peru, and Vietnam found that stunting in early childhood was associated with lower cognitive achievement in 5-year-old children (44). In infants and young children in Kenya, stunting is also associated with developing severe respiratory syncytial virus-associated pneumonia, and with lower respiratory tract infections from all causes (55).

The effect of under-nutrition on malaria outcomes is disputed in the literature. In a Kilifi HDSS study, Berkley et al. (56) found that malnutrition was associated with severe disease due to falciparum malaria. A cohort study at Farafenni HDSS in Gambia calculated that 51% of children with stunting subsequently experienced malaria during the malaria season, compared with 38% of children who were not stunted. The study authors concluded: ‘Our findings suggest that chronically malnourished children may be at higher risk for developing malaria episodes’ (34). On the other hand, Muller et al. (18), working at Nouna HDSS in Burkina Faso, found no association between protein-energy malnutrition and malaria morbidity, while a Kilifi HDSS study concluded that children with iron deficiency were less likely than other children to develop mild clinical malaria (32). Table 4 displays key findings on effects of malnutrition.

**Table 4:** Effects of malnutrition: findings from INDEPTH studies

HDSS site	Year	Authors	Main findings
Kilifi HDSS, Kenya	2008	Bejon et al.	Under-nutrition a factor in half of in-hospital deaths of children and half of morbidity from severe diseases.
West Kiang HDSS, Gambia	2011	Ngom et al.	Ten times higher mortality among young adults born in the hunger season than in harvest season.
Niakhar HDSS, Senegal	2006	Enel et al.	Decline in child mortality despite no improvement in nutritional status among children.
Niakhar HDSS, Senegal	2004	Pinchinat et al.	Decline in child mortality despite no improvement in nutritional status among children.
Matlab HDSS, Bangladesh	2008	Bosch et al.	Stunting in early childhood a significant predictor of stunting in adolescence.
Multiple sites in Ethiopia, India, Peru, Vietnam	2013	Barnett et al.	Stunting in early childhood associates with lower cognitive achievement among 5-year-olds.
Kilifi HDSS, Kenya	2008	Okiro et al.	Stunting in infants and children associated with developing severe respiratory syncytial virus-associated pneumonia, and with lower respiratory tract infections from all causes.
Kilifi HDSS, Kenya	2009	Berkley et al.	Malnutrition associated with severe falciparum malaria.
Farafenni HDSS, Gambia	2002	Deen et al.	51% of children with stunting subsequently experienced malaria during the malaria season, compared with 38% of children who were not stunted.
Nouna HDSS, Burkina Faso	2003	Muller et al.	No association between protein-energy malnutrition and malaria morbidity.
Kilifi HDSS, Kenya	2004	Nyakeriga et al.	Children with iron deficiency were less likely than other children to develop mild clinical malaria.

### Tackling malnutrition

#### The role of breastfeeding

Nutrition in infancy, as we have seen, is important to an individual's prospects of a good life. Breastfeeding has therefore been the subject of much analysis by nutrition researchers, who have assessed its benefits and analysed breastfeeding practices in LMICs.

Working at the Kintampo HDSS in Ghana, Edmond et al. (57) demonstrated for the first time that there is a causal link between early breastfeeding and declines in infection-specific neonatal mortality in infants aged between 2 and 28 days. Delayed initiation of breastfeeding until after the first day of life led to a 2.6 times higher risk of neonatal mortality as a result of infectious disease, while partial breastfeeding led to a 5.7 times higher risk. Researchers at Bandim HDSS in Guinea-Bissau found that children weaned early for reasons other than ‘being healthy’, including a new pregnancy, had higher mortality (58). Bahl et al. (59), meanwhile, found that non-breastfed infants had a 10-fold higher risk of dying than predominantly breastfed and exclusively breastfed infants, although there was no significant difference in risk between the latter two groups. This finding suggests that in areas where most women already predominantly breastfeed, breastfeeding promotion efforts should be directed at those who chose not to breastfeed or who partially breastfed. A case–control study by Saloojee et al. in rural South Africa (17) also found that risk factors for severe malnutrition included poor weaning practices.

The positive effects of breastfeeding have also been demonstrated in older children. Vohr et al. (60) found that extremely low birth weight babies who ingested breast milk while in the neonatal intensive care unit (NICU) had significantly higher scores in cognitive and behaviour tests at 18 months of age than similar non-breastfed NICU infants.

However, breastfeeding is not universally practised. Researchers at Nanoro HDSS in Burkina Faso who conducted a multi-centre study of women infected with HIV, highlighted the size of the task facing those engaged in breastfeeding promotion efforts. In sub-Saharan Africa, fluids other than maternal milk are often introduced within the first six months of life, notwithstanding WHO guidelines that recommend exclusive breastfeeding for the first six months (including where the mother is HIV-positive). Although 53% of women in the Nanoro study saw exclusive breastfeeding as the preferred feeding method, only 11% reported exclusively breastfeeding after previous pregnancies (61). Conversely, some mothers choose to prolong breastfeeding beyond the recommended duration, which has been found to be associated with reduced nutritional status and impaired growth. However, as Simondon and Simondon (62) and Simondon et al. (63) found in two studies conducted in Senegal, these consequences are a result not of prolonged breastfeeding itself, but of the fact that women prolong breastfeeding beyond 12 months for children who are undernourished and tend to wean well-nourished children. The authors surmised that this is a result of mothers’ awareness of the mortality risks to children following weaning.

#### Prevention efforts

A number of interventions to prevent or cure malnutrition have been tested by HDSS sites. The vast majority of the trials have focused on infants, children and women of childbearing age.

Efforts to tackle malnutrition in infants and children have met with mixed success. Studies by the Bandim HDSS in Guinea-Bissau of malnutrition campaigns during that country's 1998 civil war found that vitamin A supplementation for children (64) and a supplementary feeding programme for children (65) helped to reduce mortality during the conflict. Compared with the 3-year period before the war, the authors reported that children offered vitamin A at home during the war had a 12% reduction in mortality, whereas the overall impact of the war was an 89% increase in mortality.

In a randomised controlled trial in Vietnam, fortification with multiple micronutrients of biscuits provided in schools was found to reduce the risk of anaemia and of iron and zinc deficiencies by 40% (66). At Nouna HDSS in Burkina Faso, on the other hand, a randomised controlled trial to assess the effects of zinc supplementation on the growth of young children in an area with high rates of malnutrition found no significant impact on height-for-age, weight-for-age, and weight-for-height scores. The study authors concluded that zinc supplementation does not have an effect or public health importance on growth in West African populations of young children with a high prevalence of malnutrition, and that multi-nutrient interventions are likely to be more effective (67).

The effectiveness of vitamin A supplementation has also been questioned. Although it appeared to be beneficial in the above-mentioned trial in Guinea-Bissau, researchers at Kintampo HDSS in Ghana found no positive effect of vitamin A supplements in combination with vaccines during the first few months of life (68). This finding echoes the findings of similar trials in Peru and India. Fish oil supplementation, too, appears to have limited effects for infants. A study in Gambia found that fish oil had no effect on growth, intestinal integrity, morbidity, or cognitive development (69).

Only two studies investigated methods to treat children suffering from severe malnutrition. Current guidelines for treating children who develop shock as a result of severe malnutrition recommend using low-dose hypotonic fluid resuscitation. Working at the Kilifi HDSS in Kenya, Akech et al. (70) tested the safety and efficacy of this recommendation and found ‘universally poor’ outcomes in terms of persistent shock, oliguria, and high case fatality. The authors concluded that the low recommended dose was insufficient to correct shock and suggested that for future guidelines to be more effective, clinical investigation is needed to determine the appropriate volume and rate of dosage of isotonic fluids. Researchers at Agincourt HDSS, meanwhile, found that the introduction of WHO guidelines in two hospitals strengthened the management of malnutrition. The authors concluded that implementation of WHO guidelines on severe malnutrition was feasible, affordable, and sustainable (71).

Studies of malnutrition prevention campaigns for women of childbearing age also had mixed results. In Ghana, vitamin A supplementation was found to increase liver reserves of mothers during the postpartum period, with the effects persisting for at least five months (72). A study at Niakhar HDSS in Senegal found that providing high energy, nutrient-dense food supplements to infants aged between four and seven months contributed to decreased postpartum weight loss in their mothers (73). The supplements reduced the intake of breast milk and therefore the energy costs of lactation on mothers. It also increased the interval between births, thereby potentially having effects on fertility. As with supplements for children, a number of studies – all conducted at the West Kiang HDSS in Gambia – found that supplements of individual nutrients for women were ineffective. Hawkesworth et al. (74) found that nutrient supplementation during pregnancy had little effect on infants’ risk of cardiovascular disease. Another study found that protein-energy supplementation during pregnancy had no effect on the blood pressure of their children in adolescence (75), while a third study found that calcium supplementation for pregnant mothers had no effect on foetal and infant growth (21). A fourth West Kiang study found that calcium supplements could be harmful – women given calcium supplements while pregnant were found to have lower bone mineral content, bone area, and bone mineral density at the hip during the 12-month lactation period than other women. The authors surmised that such supplements may disrupt metabolic adaptation (76).

Two studies conducted by INDEPTH members in South Africa and Bangladesh addressed the problem of implementing nutrition programmes. In the first, at Dikgale HDSS, the majority of people with type 2 diabetes had poor glycaemic control and were obese or had high blood pressure. Quantitative and qualitative research showed that these patients were often given incorrect and inappropriate dietary advice by health educators (77). In the second study, researchers showed that agencies working to tackle a flood disaster in Bangladesh in 1998 had mixed results in terms of meeting international disaster response standards. Compliance with standards was variable. Preliminary nutritional analysis was one of the areas in which agencies performed poorly in this regard (78).

## Discussion

### The state of research

Over the past 15 years, INDEPTH member centres have expanded the global knowledge base on nutrition. They have shown how under-nutrition continues to plague children and adults of all ages in LMICs and they have highlighted the emergence of obesity as a growing threat. In some areas, combined under-nutrition early in life with overweight and obesity in adolescence and adulthood, warns of rapidly escalating risk for cardiometabolic disease. The HDSS centres have demonstrated how malnutrition early in life continues to have effects on physical and mental development throughout the life-cycle. They have examined links between disease and malnutrition, and shown how wealth, education, and urbanisation all reduce the risks of under-nutrition but at the same time can increase the risks of obesity. The double burden of malnutrition in LMICs is of major concern to the World Bank, the WHO, and other international bodies.

In terms of tackling malnutrition, INDEPTH members have demonstrated the importance of breastfeeding for infant nutrition and health, and for the health of the infants as they grow older, and they have assessed the reasons why some women choose not to breastfeed or to wean infants too early or too late. Interventions such as micronutrient supplementation for children and their mothers can have beneficial impacts on child health and survival, with some studies suggesting that multiple micronutrient supplementation may be more effective than supplementation with single micronutrients (Table 5).

**Table 5:** Tackling malnutrition – prevention efforts: findings from INDEPTH studies

HDSS site	Year	Authors	Main findings
Bandim HDSS, Guinea-Bissau	2004	Nielsen et al.	During civil war, vitamin A supplementation for children helped reduce mortality.
Bandim HDSS, Guinea-Bissau	2005	Nielsen et al.	During civil war, supplementary feeding programme helped reduce mortality in children.
FilaBavi HDSS, Vietnam	2009	Nga et al.	Fortification with multiple micronutrients of biscuits provided in schools reduced risk of anaemia and of iron and zinc deficiencies by 40%.
Nouna HDSS, Burkina Faso	2003	Müller et al.	No significant impact of zinc supplementation for children on height-for-age, weight-for-age, and weight-for-height scores.
Kintampo HDSS, Ghana	2007	Newton et al.	No positive effect of vitamin A supplements in combination with vaccines during the first few months of life.
Farafenni HDSS, Gambia	2013	Van der Merwe et al.	Fish oil supplementation had no effect on growth, intestinal integrity, morbidity, or cognitive development.
Kilifi HDSS, Kenya	2010	Akech et al.	Use of low-dose hypotonic fluid resuscitation for children who develop shock as a result of severe malnutrition has poor outcomes in terms of persistent shock, oliguria, and high case fatality.
Agincourt HDSS, South Africa	2003	Deen et al.	Introduction of WHO guidelines in two hospitals strengthened the management of malnutrition.
Kintampo HDSS, Ghana	2006	Tchum et al.	Vitamin A supplementation increased liver reserves of mothers during the postpartum period, with effects persisting for at least five months.
Niakhar HDSS, Senegal	2006	Ly et al.	Provision of high energy, nutrient-dense food supplements to infants aged between four and seven months contributed to decreased postpartum weight loss in their mothers.
West Kiang HDSS, Gambia	2011	Hawkesworth et al.	Nutrient supplementation during pregnancy had little effect on infants’ risk of cardiovascular disease.
West Kiang HDSS, Gambia	2009	Hawkesworth et al.	Protein-energy supplementation of mothers during pregnancy had no effect on the blood pressure of their children in adolescence.
West Kiang HDSS, Gambia	2009	Prentice et al.	Calcium supplementation for pregnant mothers had no effect on foetal and infant growth.
West Kiang HDSS, Gambia	2010	Jarjou et al.	Women given calcium supplements while pregnant had lower bone mineral content, bone area and bone mineral density at the hip during the 12-month lactation period than other women.
Dikgale HDSS, South Africa	2002	Nthangeni et al.	The majority of people with type 2 diabetes had poor glycaemic control and were obese or had high blood pressure and were given poor advice by health educators.
Africa Centre HDSS, South Africa	2002	O'Donnell et al.	Agencies working to tackle a flood disaster in Bangladesh in 1998 achieved mixed results in terms of meeting international disaster response standards. Some agencies complied with most standards, some with few.

Nutrition is something of an under-researched field; however, over the period 1999–2013, INDEPTH member centres produced almost four times as many published papers on HIV/AIDS as they did on nutrition. Many issues remain unresolved – for example, the impact of malaria on malnutrition and of malnutrition on susceptibility to malaria. There was little attention paid to nutrient supplements or to education and information campaigns to help families improve their diet, while activism campaigns to encourage companies to label foods accurately or to reduce sugar or fat content received no attention from HDSS centres. Likewise, the economic benefits of nutrition interventions also received little attention from HDSSs. One study by the Africa Centre calculated the costs of adding nutritional supplementation to the management of HIV-infected children (79), but studies on the cost-effectiveness of nutrition programmes were lacking.

### Policy implications

In this review, we noted the importance of adopting a multi-pronged approach to tackling malnutrition. Countries including Vietnam and South Africa have had some success in reducing malnutrition in recent years. Researchers at Kaya HDSS in Burkina Faso carried out a nine-site study to assess the impact of United Nations Millennium Project guidelines to reduce child stunting. This holistic approach incorporated nutrition-specific, health-based interventions with food system and livelihood-based approaches, and its adoption reduced stunting in children by up to 43% over 3 years (80). Programmes in Vietnam to increase nutrition, particularly via animal-source foods, have also been successful in reducing under-nutrition in children and chronic energy deficiency in women (81), but otherwise investigations of nationwide or multi-pronged nutrition programmes have not been extensively studied by HDSS centres. Lessons from other countries on how to reduce malnutrition may be a useful guide for policy-makers who are attempting to tackle the problem in their own countries.

Although a small number of studies have examined the success of efforts to implement nutrition programmes, there is a need for further investigation in this area. How to effectively change dietary habits, how to educate individuals in LMICs about the availability of healthy natural foods, how to improve compliance with food supplementation programmes, and how to train health workers to provide useful nutrition advice and effective treatment for nutrition problems, are just a few of the many implementation challenges that remain.

The INDEPTH HDSSs have unearthed important findings over the past decade and a half – for example, the vital role of breastfeeding in promoting infant and child health, the benefits of multiple micronutrient supplementation, the under-appreciated prevalence of malnutrition in adults and the elderly, and the rise of obesity in lower-income settings. Although much knowledge generation has already been achieved, there is broad scope for future research to inform policy-makers and researchers on ways to tackle the double burden of malnutrition in order to have a major impact on policy and on the lives of people living in LMICs.
